# Association between ABO blood group system and autoimmune liver disease

**DOI:** 10.3389/fmed.2025.1696577

**Published:** 2025-11-17

**Authors:** Yi Hong, Shijie Mu, Lin Wang

**Affiliations:** 1 Department of Transfusion Medicine, Xi'an Gaoxin Hospital, Xi’an, China; 2 Department of Transfusion Medicine, Tangdu Hospital, The Fourth Military Medical University, Xi’an, China

**Keywords:** ABO blood type, autoimmune liver disease, autoimmune hepatitis, primary biliary cholangitis, case control study

## Abstract

**Background:**

Autoimmune liver disease (AILD) is a chronic inflammatory liver disease mediated by abnormal autoimmune response, and its pathogenesis has not been fully elucidated. Studies have shown that ABO blood is associated with autoimmune diseases, but the relationship between ABO blood groups and AILD has not been studied. The purpose of this study is to examine whether there is a correlation between ABO blood group and AILD.

**Methods:**

A retrospective cohort study was conducted on 114 AILD patients and 1,167 healthy individuals from January 2019 to February 2024. Blood type was determined with the gel microcolumn assay. Chi-square test and logistic regression were performed to investigate the association between blood type and the risk of AILD.

**Results:**

Of 114 patients, 44 (38.60%) were diagnosed with autoimmune hepatitis (AIH), and 70 (61.40%) were diagnosed with primary biliary cholangitis (PBC). A significant difference was found between ABO blood groups and AILD patients. Compared with the controls, blood type A was more prevalent in the AILD group (OR, 1.784; *p* < 0.05), whereas blood type B was less (OR, 0.638; *p* < 0.05). Moreover, patients with PBC had a higher percentage of type A (OR, 2.656; *p* < 0.01) and a lower percentage of type B (OR, 0.514; *p* < 0.05). No significant difference was found in blood type O and AB (*p* > 0.05).

**Conclusion:**

Our findings suggest that ABO blood groups could potentially affect the presence of AILD.

## 1 Introduction

ABO is the most important blood group system in humans, which is generally divided into four types according to the A and B antigens contained in human red blood cells (RBCs): A, B, AB and O. A large number of researches have shown that ABO blood group is related to many diseases, including cancer, vascular diseases, gastroduodenal ulcers, severe Plasmodium falciparum malaria and diabetes (1). At present, there are few study results on the relationship between ABO blood group and the risk of autoimmune diseases in different populations, and the results are not consistent. Nik et al. (2) explored the distribution of ABO and Rh blood groups in systemic lupus erythematosus (SLE) and rheumatoid arthritis (RA) patients and their relationship with disease manifestations, and found no difference in the distribution of blood groups in RA and SLE patients. Salem et al. (3) investigated the association between ABO and Rh blood groups and the type of RA, and the results showed that A and Rh-positive blood groups were more frequent in patients with RA. Those interesting findings point to the possible interference between the pathway of blood group production and auto-antibodies.

Autoimmune liver disease (AILD) is a chronic disease caused by an immune-mediated auto-aggressive inflammatory response in genetically susceptible individuals. There are several forms, including autoimmune hepatitis (AIH), primary biliary cholangitis (formally known as cirrhosis, PBC), and primary sclerosing cholangitis (PSC) (4). Epidemiological statistics indicate that the incidence and prevalence of AILD are increasing all over the world (5). Studies have shown that AILD is related to genetic and environmental factors and abnormal immune tolerance, and individuals with genetic susceptibility may produce abnormal immune responses under the action of environmental factors, resulting in autoimmune-mediated liver cell damage (6). Indeed, the presenting clinical characteristics of this disease are non-specific. Jaundice, digestive malabsorption, malnutrition, anemia, bleeding, and fever are the dominant clinical features.

Due to the correlation between ABO blood type and the production of autoimmune antibodies, it may also affect the pathogenesis of AILD. However, there are few studies on the correlation between ABO blood group and AILD. Therefore, this study aims to retrospectively analyze the clinical data of AILD patients in China to explore whether there is a correlation between ABO blood group and the incidence of AILD, so as to provide some evidence for clinical diagnosis and treatment of AILD.

## 2 Methodology

### 2.1 Subjects

In this retrospective, single-center cohort study, 114 patients with AILD and 1,167 healthy controls were selected from January 2019 to February 2024 in Tangdu Hospital. The patients with AILD were diagnosed according to the International Autoimmune Hepatitis Group scoring system (7). And the gender and age were recorded for each patient.

The inclusion criteria consisted of: (1) Fulfilling the definition of AILD; (2) Having complete clinical records; and (3) Conducting an adequate clinical examination. And the exclusive criteria included: (1) Patients with decompensated liver cirrhosis; (2) Co-infection with HAV, HIV, CMV, HBV, HDV, and HCV; (3) Presence of serious mental and physical diseases, including uncontrolled primary kidney, heart, lung, vascular, nervous, digestive, metabolic and immune deficiencies, etc.; and (4) Suffering from malignant tumors or having a history of malignant tumors.

### 2.2 Data collection

The clinical data of the subjects were obtained from the hospital information system of the Tangdu Hospital, including gender, age, education level, ABO blood group, and AILD types. ABO blood group identification was carried out according to the instructions of blood group gel card (Jiangsu Libo Medicine Biotechnology Co., Ltd., Jiangsu, China), and the reports were collected through the laboratory information management system.

### 2.3 Variables

In this study, 4 variables were identified (age, gender, ABO blood group, and AILD types). Age was classified as <17 years, 17–35 years, 35–55 years, and >55 years. And the ABO blood group system consisted of A, B, O and AB, which was characterized by the presence of A and B antigens on the surface of RBCs. In addition, AIH was diagnosed according to the revised and simplified International Autoimmune Hepatitis Group (IAIHG) scoring system. And PBC was diagnosed if at least two of the following three criteria were met: (1) chronic elevation of cholestatic liver enzymes alkaline phosphatase and gamma-glutamyl transpeptidase for at least 6 months; (2) the presence of serum antimitochondrial antibodies; and (3) typical histological findings in biopsied liver specimens.

### 2.4 Statistical analysis

Microsoft Excel 15.19.1 and IBM SPSS statistics 22.0 (SPSS Inc., Chicago, United States) were used to analyze the data. Categorical variables were presented as actual numbers and percentages. Chi-squared test or Fisher’s exact test was performed to compare the group of variables. The logistic regression analysis was conducted to identify the relationship between AILD and the distribution of ABO blood types, while the odds ratio (OR) and 95% confidence interval (CI) were calculated. All tests were carried out using a bilateral test, and *p* < 0.05 was considered statistically significant.

## 3 Results

### 3.1 The characteristics of patients

A total of 114 patients with AILD, and 1,167 healthy people were enrolled in this study. Among them, there were 19 (16.67%) males and 95 (83.33%) females in patients with AILD and 660 (56.56%) males and 507 (43.44%) females in the control group. The age distribution of patients ranged from 18 to 84 years old, and that of healthy controls in the range of 1 to 96 years old. Significant differences were found in gender and age between patients with AILD and healthy controls (*p* < 0.01) (Table 1).

**Table 1:** Gender and age distribution of subjects.

Characteristics	AILD	Control group	χ^2^	OR	*P*
Gender					
Male	19 (16.67%)	660 (56.56%)	66.337	6.509	<0.01
Female	95 (83.33%)	507 (43.44%)			
Age					
<17	0 (0%)	60 (5.14%)	24.785	1.898	<0.01
17–35	4 (3.51%)	212 (18.17%)			
35–55	40 (35.09%)	354 (30.33%)			
>55	70 (61.40%)	541 (46.36%)			

OR, odds ratio.

### 3.2 Distribution of ABO blood types between two groups

The distribution of ABO blood groups in the control group and patients with AILD was shown in Table 2. The results indicated that the distribution of ABO in the case group was A (38.60%), B (23.68%), O (27.19%) and AB (10.53%). And the control group was A (26.05%), B (32.73%), O (29.82%), and AB (11.40%). It was observed that there was a significant difference in the distribution of ABO blood types (2 = 8.994, *p* < 0.05).

**Table 2:** Distribution of ABO blood types between two groups.

Blood group	AILD	Control group	χ^2^	*P*
A	44 (38.60%)	304 (26.05%)	8.994	0.029
B	27 (23.68%)	382 (32.73%)		
O	31 (27.19%)	348 (29.82%)		
AB	12 (10.53%)	133 (11.40%)		

OR, odds ratio.

**Figure 1:**
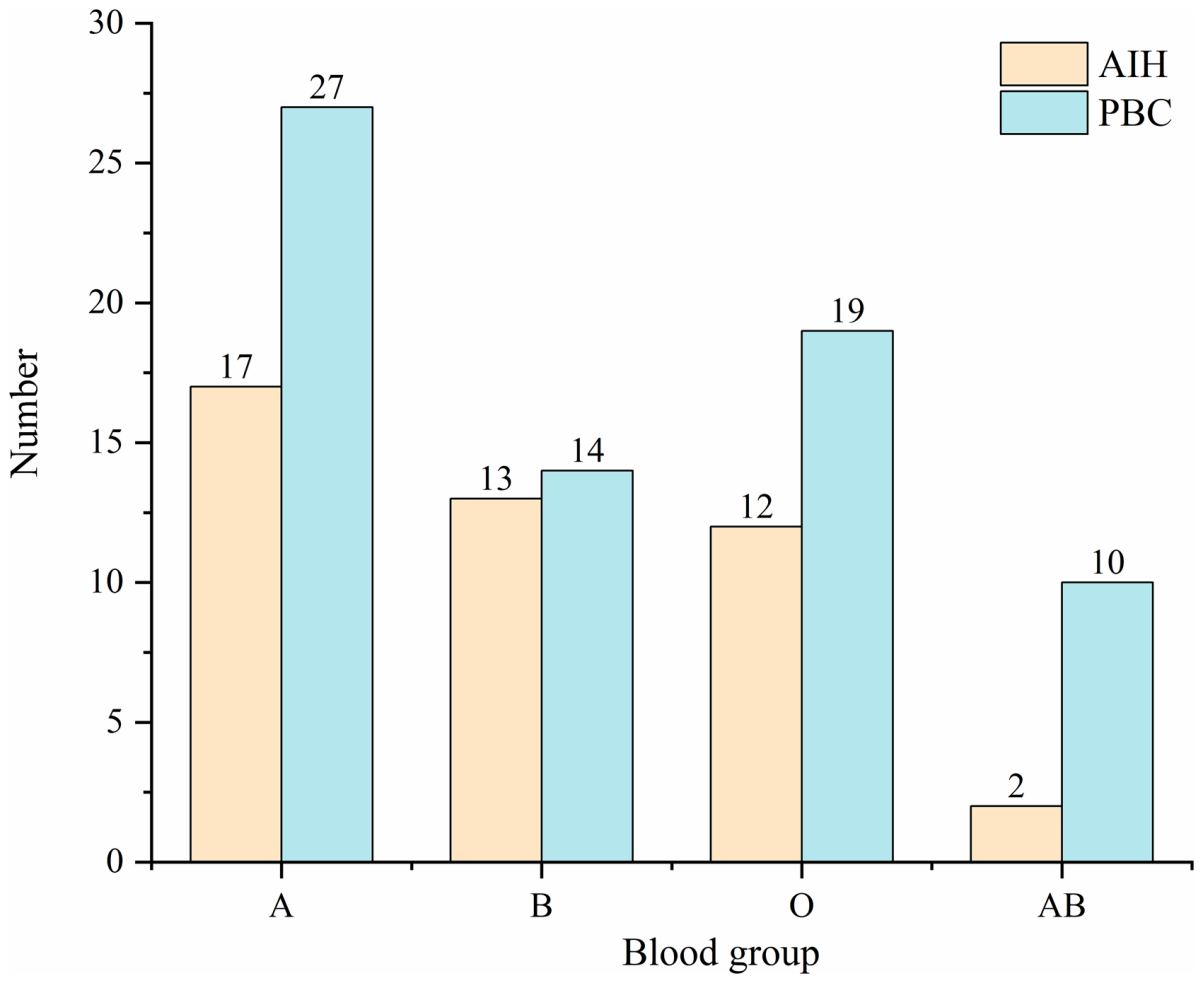
Frequency of distribution of ABO blood groups in AIH and PBC.

In addition, among all patients, 44 (38.60%) were diagnosed with AIH, and 70 (61.40%) were diagnosed with PBC. The most frequent blood groups of the AIH patients were as follows: A (38.64%), B (29.54%), O (27.27%), and AB (4.55%). And the distribution of ABO blood types in PBC patients was A (38.57%), B (20.00%), O (27.14%), and AB (14.29%) (Figure 1). Compared with healthy controls, there was no statistical difference in the distribution of ABO blood groups in AIH patients, but there was a statistical difference in PBC patients.

### 3.3 The association between ABO blood types and the AILD

In order to investigate the effect of ABO blood types in the AILD, logistic regression analysis was carried out. And the results indicated that blood type A was more frequent in AILD patients (OR, 1.784; *p* < 0.05). In contrast, blood type B was less common in the case group (OR, 0.638; *p* < 0.05). There was no significant difference was found in blood type O and AB (Table 3).

**Table 3:** Odds of AILD according to ABO blood types.

Blood group	AILD	Control group	χ^2^	OR	95%CI	*P*
A	44 (38.60%)	304 (26.05%)	8.263	1.784	1.197–2.660	0.004
Non-A	70 (61.40%)	863 (73.95%)				
B	27 (23.68%)	382 (32.73%)	3.913	0.638	0.407–0.999	0.048
Non-B	87 (76.32%)	785 (67.27%)				
O	31 (27.19%)	348 (29.82%)	0.344	0.879	0.571–1.353	0.558
Non-O	83 (72.81%)	819 (70.18%)				
AB	12 (10.53%)	133 (11.40%)	0.078	0.915	0.490–1.708	0.779
Non-AB	102 (89.47%)	1,034 (88.60%)				

OR, odds ratio; CI, confidence interval.

Moreover, the results of ABO blood group distribution analysis in PBC patients showed that blood type A had a connection with increased incidence of PBC (OR, 2.656; *p* < 0.05), whereas those with blood type B might be less susceptible to PBC (OR, 0.514; *p* < 0.05). Type O and type AB were not statistically significant between patients with PBC and controls (Table 4).

**Table 4:** Odds of PBC according to ABO blood types.

Blood group	PBC	Control group	χ^2^	OR	95%CI	*P*
A	27 (38.57%)	304 (26.05%)	5.283	2.656	1.603–4.401	< 0.01
Non-A	43 (61.43%)	863 (73.95%)				
B	14 (20.00%)	382 (32.73%)	4.920	0.514	0.282–0.934	0.027
Non-B	56 (80.00%)	785 (67.27%)				
O	19 (27.14%)	348 (29.82%)	0.227	0.877	0.510–1.507	0.634
Non-O	51 (72.86%)	819 (70.18%)				
AB	10 (14.29%)	133 (11.40%)	0.539	1.296	0.648–2.592	0.463
Non-AB	60 (85.71%)	1,034 (88.60%)				

OR, odds ratio; CI, confidence interval.

In addition, multivariate analysis showed that age, gender, and the ABO blood group were independent prognostic factors (Table 5).

**Table 5:** Multivariate analysis of AILD.

Characteristics	OR	95%CI	*P*
Gender	37.016	14.096–97.205	<0.01
Age	3.161	1.601–6.238	<0.01
Blood group	0.093	0.059–0.146	<0.01

OR, odds ratio; CI, confidence interval.

## 4 Discussion

The ABO blood group type is identified by the expression of A, B or H antigens of carbohydrate epitopes on the surface of RBCs and glycolipids (8). Blood group antigens are initially thought to exist only on the surface of RBCs, and the research on them has mainly focused on serology. Subsequent studies have found that blood group antigens can be widely expressed in other tissues and body fluids outside RBCs in the form of glycoproteins, glycolipids and free oligosaccharides (9). Studies have shown that in addition to playing a role in blood transfusion and blood typing, blood group antigens can also be used as direct receptors of signal molecules on the surface of cell membranes, and participate in important life processes such as cell recognition, adhesion, intercellular signal transduction, immune response, inflammation, autoimmune diseases, aging, abnormal proliferation and metastasis of cancer cells, as well as pathogen infection (10, 11). Karimifar et al. (12) discovered that the severity of SLE in blood groups A and B was higher than in the other two blood groups. Chen et al. (13) revealed that there was a significant correlation between blood type and gastric non-cardiac adenocarcinoma, and the risk of type A and AB was higher than that of type O. Hoiland et al. (14) determined whether ABO blood groups were associated with different severities of COVID-19, and results showed that critically ill COVID-19 patients with blood group A or AB were at increased risk for requiring mechanical ventilation, CRRT, and prolonged ICU admission compared with patients with blood group O or B.

In this study, we found that the most frequent blood type in patients with AILD was the A blood group, followed by O, B, and AB, respectively. And there were significant differences in the distribution of type A and type B between patients with AILD and healthy individuals. No significant difference was detected in the distribution of type O and type AB. The susceptibility of type A was the highest and that of type B was the lowest.

Moreover, our study additionally investigated the ABO blood type distribution of different types of AILD, and the results indicated that the blood group distribution of AIH was not statistically significant compared to the control group, while that of PBC was. The distribution of blood type A in PBC was higher than that of blood type AB and O, and the difference was statistically significant. Therefore, it could be said that people with blood type A were more likely to develop PBC than people with blood types AB and O. There was an opposite situation for blood type B compared to blood types AB and O, meaning that type B blood was less likely to develop PBC than the two groups above.

**Figure 2:**
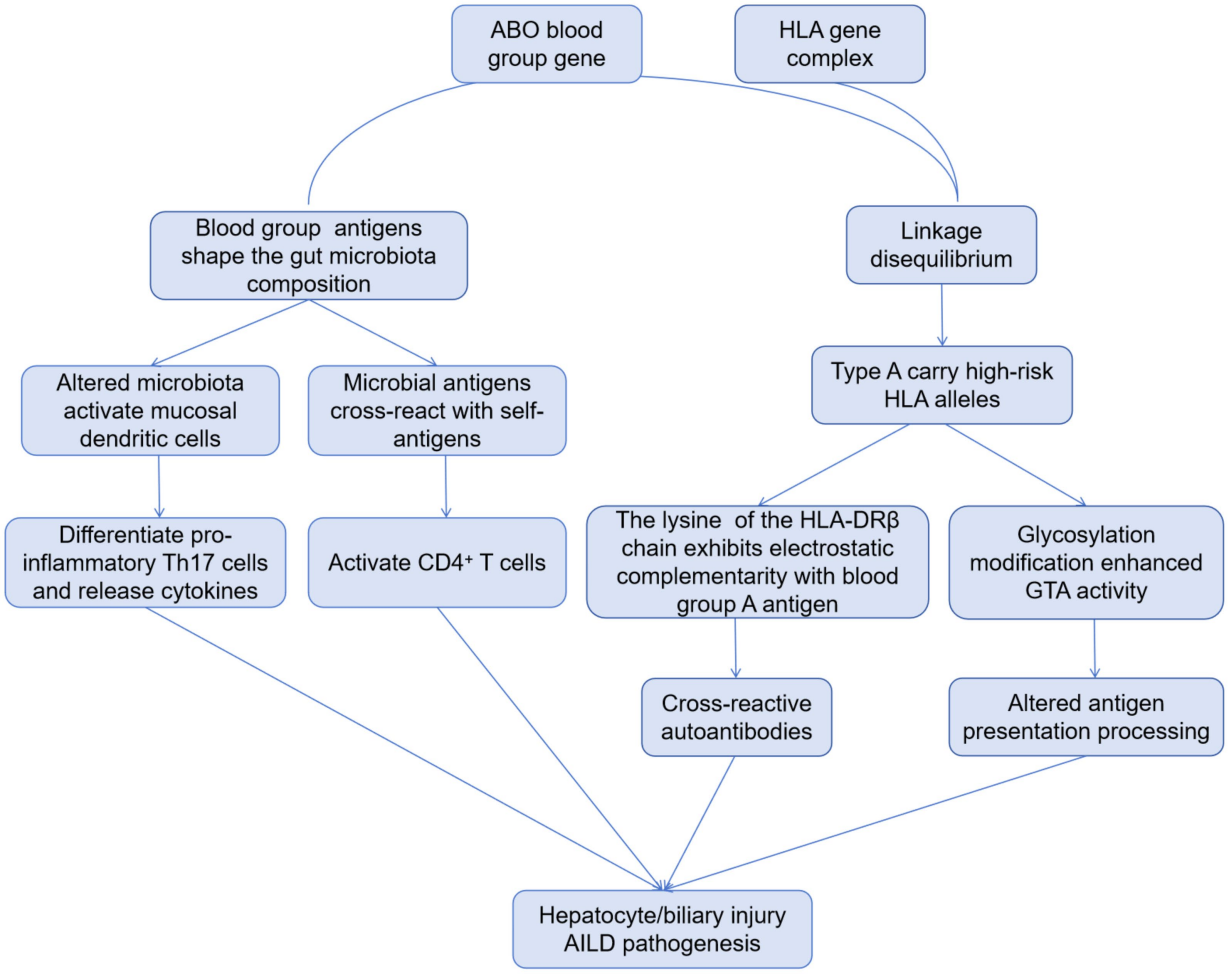
Proposed mechanistic pathways linking ABO blood group to AILD.

It has been confirmed that human leukocyte antigen (HLA) class I and II-haplotypes are strongly related to the pathogenesis and development of AILD (15). Although the ABO blood group gene (9q34.2) and the HLA complex (6p21.3) are located on different chromosomal regions, they both belong to immune-related gene clusters and have developed significant linkage disequilibrium (LD) during evolution due to genetic drift or selective pressure. The association between type A blood and AILD discovered in this study may essentially be that individuals with type A blood “carry” multiple high-risk HLA alleles for AILD through LD. Studies have shown that there is a significant linkage disequilibrium between ABO blood type (especially type A) and HLA-DRB1 and DQB1 alleles (for example, people with type A blood are more likely to carry HLA-DRB1*03:01 and DRB1*04:01 alleles) (16). These HLA alleles have been confirmed to be associated with AILD susceptibility (for example, HLA-DRB1*03:01 is associated with AIH and PBC; DRB1*04:01 is associated with PBC) (17). Therefore, people with blood type A carry high-risk HLA alleles due to LD, which constitutes an important basis for genetic susceptibility to AILD. Moreover, the lysine at position 71 of the HLA-DRβ chain (the characteristic residue of DRB1*03:01) exhibits electrostatic complementarity with the terminal N-acetylgalactosamine of blood group A antigen (18, 19). This structural similarity may lead to aberrant activation of B cells targeting blood group A antigens, subsequently inducing the production of cross-reactive autoantibodies that attack hepatocytes (20). Meanwhile, the ABO gene encodes glycosyltransferases that modify N- or O-linked glycans on cell surface glycoproteins (21). Due to the activity of *α*-1, 3-N-acetylgalactosaminotransferase (GTA) in individuals with type A blood, specific glycosides (such as A antigen) may be introduced into the glycosylation modification of HLA molecules, affecting their binding efficiency with antigen processing associated transporters (TAP), or altering the loading preference of antigen peptides, making it easier for self-antigen peptides to be presented. Besides, ABO blood group antigens are widely expressed in intestinal mucosal epithelium and secretions, and can indirectly regulate immune homeostasis by shaping the colonization environment of the microbiota (22, 23). The A antigen on the intestinal mucosa surface of individuals with type A blood can act as an adhesion receptor for symbiotic bacteria (such as Bacteroides), promoting the enrichment of specific bacterial communities (such as sulfate-reducing bacteria) (24). On the one hand, these microbiota can induce the differentiation of Th17 cells by activating intestinal mucosal dendritic cells (DCs), promote the secretion of inflammatory factors (IL-17A, IL-22), and enhance liver immune infiltration (25). On the other hand, microbiota antigens (such as lipopolysaccharides and peptidoglycan) may have cross-reactions (molecular simulation) with autoantigens, activating CD4^+^ T cells targeting liver autoantigens through the cross-presentation of HLA class II molecules (26). Collectively, the association between blood type and elevated risk for AILD can be conceptualized through several interconnected immunological pathways, encompassing HLA-mediated genetic predisposition, cross-reactive autoantibody induction, and microbe-dependent immunomodulation, which are schematically summarized in Figure 2.

Furthermore, we also discovered that females were more susceptible to AILD than males, and the reasons for this gender difference might be determined by sex hormones, fetal microchimerism, and genetic factors, which was consistent with published research results (27). In addition, the majority of AILD patients were older than 55 years old, followed by 35 to 55 years old, 17 to 35 years old, and none under 17 years old, showing that the incidence was increasing with age.

Our study has some limitations. First, it was a single-center, retrospective and observational study with a relatively small sample size, the findings of this research need to be validated in a larger-scale, multi-center prospective cohort. Second, the age distribution was different between patients with AILD and healthy controls. Due to AILD is infrequent, we could only retrospectively enroll these patients. Third, due to the limitations of the research design and the scarcity of biological samples, the study failed to conduct direct experimental verification of the mechanism hypothesis, especially lacking HLA genotyping data of patients in the cohort to directly analyze the gene–gene interaction between HLA and ABO blood types.

## 5 Conclusion

In summary, we found that there was a statistical difference in the distribution of ABO blood groups in patients with AILD. The A blood group might be more prone to AILD, whereas B blood group might be less susceptible to AILD. In addition, we also revealed patients with blood group A had a significantly higher risk of developing PBC, while patients with blood group B had a significantly lower risk of developing PBC. No significant difference was found in O and AB blood groups. Our findings suggest that ABO blood groups could potentially affect the presence of AILD.

## Data Availability

The raw data supporting the conclusions of this article will be made available by the authors, without undue reservation.
